# Supplementary material for: Investigation of the incidence trend of follicular lymphoma from 2008 to 2017 in Taiwan and the United States using population-based data
Source: PLoS One. 2022 Mar 17;17(3):e0265543. doi: 10.1371/journal.pone.0265543 (PMC8929617; doi:10.1371/journal.pone.0265543)
Supplement: S1 File — (DOCX) [file pone.0265543.s001.docx]

**S1 File**

**S1 Table. Data source**

| Database | Year | Area |
| --- | --- | --- |
| TCR annual report | 2008-2017 | Taiwan.  Available from: https://www.hpa.gov.tw/Pages/List.aspx?nodeid=269 (released Jan 2021) |
| Resident Population, Taiwan | 2008-2017 | Taiwan.  Available from: https://www.moi.gov.tw/files/site_stuff/321/1/month/m1-06.ods |
| SEER 18 Regs Research Data | 2000-2017 | San Francisco-Oakland SMSA, San Jose-Monterey, Connecticut, Los Angeles, Detroit (Metropolitan), Alaska Natives, Hawaii, Rural Georgia, Iowa, California excluding SF/SJM/LA, New Mexico, Kentucky, Seattle (Puget Sound), Louisiana, Utah, New Jersey, Atlanta(Metropolitan), and Greater Georgia.  Available from: https://www.seer.cancer.gov |

Abbreviation: SEER,Surveillance, Epidemiology, and End Results

**S2 Table. Incidence rates of follicular lymphoma according to sex in Taiwan from 2002 to 2007**

| **Year** | **2002** | **2003** | **2004** | **2005** | **2006** | **2007** |
| --- | --- | --- | --- | --- | --- | --- |
| **Males** | 0.56 | 0.70 | 0.62 | 0.77 | 0.74 | 0.72 |
| **Females** | 0.41 | 0.54 | 0.51 | 0.51 | 0.59 | 0.67 |

Data were obtained from the study of Ko et al.^10^

**S3 Table. Annual percent changes in Taiwan, Korea, and Japan**

|  | **TW_M** | **TW_F** | **Korea_Lee** | **Korea_Kim** | **Japan** |
| --- | --- | --- | --- | --- | --- |
| **APC** | 2.3* | 4.1* | 5.7* | 7.9 | 12.7* |
| **95% CI** | (1.3, 3.4) | (3.0, 5.3) | (2.8, 8.7) | (−1.6, 18.4) | (9.6, 15.8) |
| ***P* value** | <0.001 | <0.001 | 0.001 | 0.079 | <0.001 |

TW_M indicates males in Taiwan from 2002 to 2017, estimated by combing data from Ko et al.^10^ and data used in the present report; TW_F, females in Taiwan from 2002 to 2017, estimated by combing data from Ko et al.^10^ and data used in the present report; Korea_Lee, overall annual percent change (APC) in Korea from 2001 to 2012 based on the study of Lee et al.^8^; Korea_Kim, overall APC in Korea from 2011 to 2015 based on the study by Kim et al.^9^; Japan, overall annual percent change in Japan from 2001 to 2008 based on the study by Chihara et al.^7^

**S1 Fig. Trends in the age-standardized incidence rates of follicular lymphoma in Taiwan, Japan, and Korea.**

**S4 Table. Annual percent changes in Taiwan stratified by age groups**

|  | **APC** | **95% CI** | ***P* value** |
| --- | --- | --- | --- |
| **Males and females** |  |  |  |
| **All age groups** | 3.2 | (1.6, 4.8) | 0.002* |
| **Age < 34** | 4.4 | (−3.5, 12.9) | 0.243 |
| **Age 35-64** | 2.6 | (0.5, 4.7) | 0.020* |
| **Ages ≥ 65** | 4.0 | (1.4, 6.7) | 0.007* |
| **Males** |  |  |  |
| **All age groups** | 2.6 | (0.3, 5.0) | 0.032* |
| **Age < 34** | 1.6 | (−6.6, 10.4) | 0.682 |
| **Age 35-64** | 2.7 | (−0.9, 6.3) | 0.124 |
| **Ages ≥ 65** | 2.9 | (−0.5, 6.4) | 0.089 |
| **Females** |  |  |  |
| **All age groups** | 3.8 | (1.4, 6.3) | 0.006* |
| **Age < 34** | 8.2 | (−2.4, 20.0) | 0.115 |
| **Age 35-64** | 2.5 | (−0.1, 5.2) | 0.060 |
| **Ages ≥ 65** | 5.5 | (1.2, 9.9) | 0.017* |
