# Supplementary figures and images for: Investigation of the incidence trend of follicular lymphoma from 2008 to 2017 in Taiwan and the United States using population-based data
Source: PLoS One. 2022 Mar 17;17(3):e0265543. doi: 10.1371/journal.pone.0265543 (PMC8929617; doi:10.1371/journal.pone.0265543)

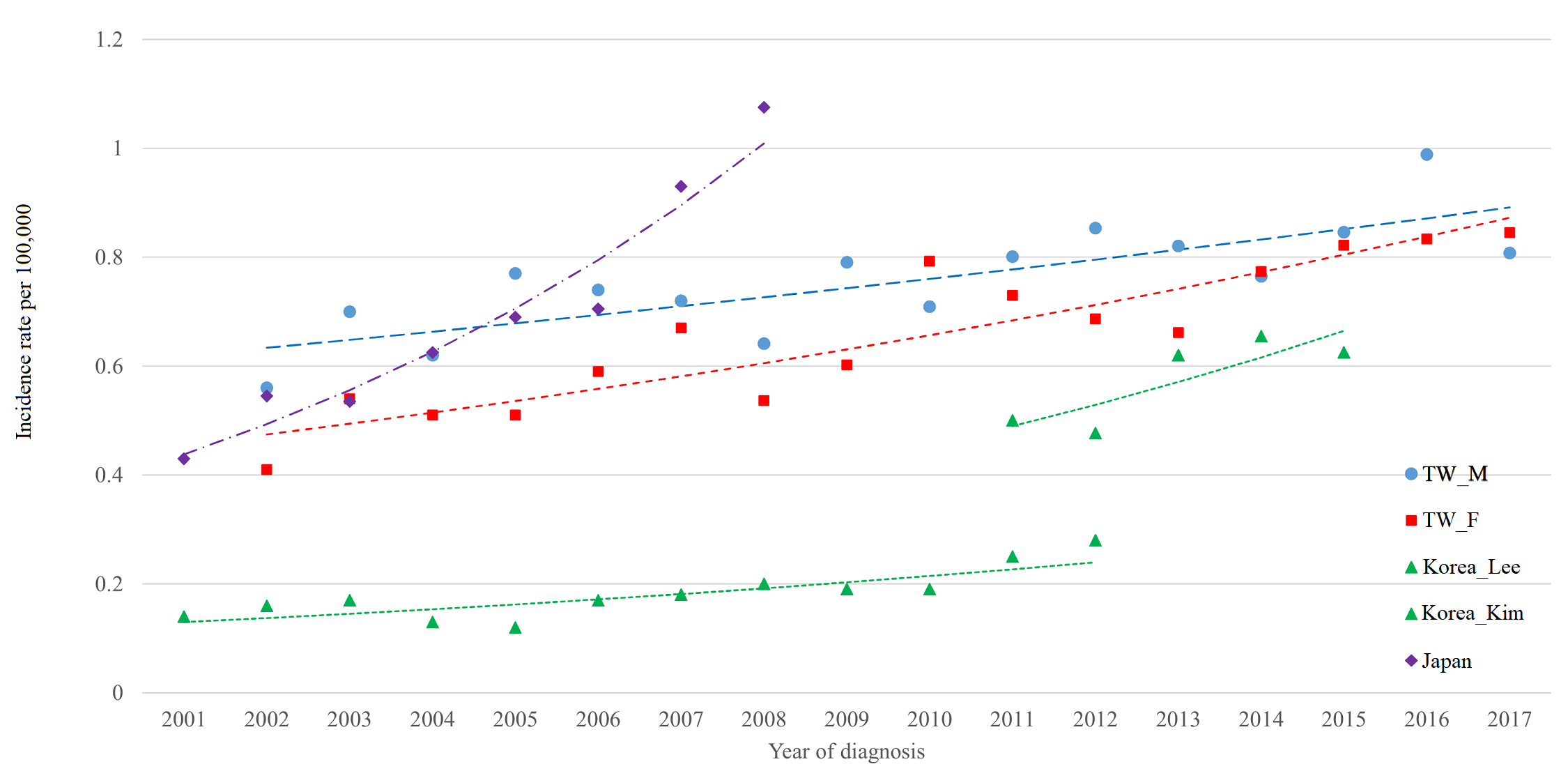

Supplement: S1 Fig — (TIF) [file pone.0265543.s002.tif]
