# Supplementary material for: Investigation of the incidence trend of follicular lymphoma from 2008 to 2017 in Taiwan and the United States using population-based data
Source: PLoS One. 2022 Mar 17;17(3):e0265543. doi: 10.1371/journal.pone.0265543 (PMC8929617; doi:10.1371/journal.pone.0265543)
Supplement: Attachment — Submitted filename: Response to Reviewers.docx [file pone.0265543.s003.docx]

To Editor and Reviewers:

Thank you very much for your detailed review of our manuscript PONE-D-21-21835, entitled “**Investigation of the incidence trend of follicular lymphoma from 2008 to 2017 in Taiwan and the United States using population-based data**”. We have provided a point-by-point revision or response to all your comments in balance. We provide the reasons and revisions in the following parts. For your convenience, all the changes are a highlight in red. During the revision, we also identify some mistakes in our article. We corrected them and marked in red.

We appreciate your valuable opinions. And thank you again for your kindly consideration for publication of our revision article.

Best regards.

Yu-Chieh Su MD, MSc.

Division of Hematology-oncology, E-Da hospital, Kaohsiung, Taiwan.

8, Oct, 2021

**Data Availability:**

- TCR annual report are available from <https://www.hpa.gov.tw/Pages/List.aspx?nodeid=269> (released Jan 2021)

- Resident Population, Taiwan are available from <https://www.moi.gov.tw/files/site_stuff/321/1/month/m1-06.ods>

- SEER 18 Regs Research Data are available from the Surveillance, Epidemiology, and End Results (SEER) database of the National Cancer Institute to any researcher who signs a SEER Research Data Agreement form. SEER data can be accessed here: <http://seer.cancer.gov/data/>

**Answer to Reviewers’ comment:**

**Revierwer #1:**

**Question** 1.
The results presented in Table 1 and Table S2 show apparent differences between males and females across the different populations. As exploratory analysis, statistical tests could be performed. For example, the difference of -1.8 in the APC between males and females in the Taiwanese population in Table S2 reveals a p-value p = 0.023, indicating a difference in the APC between males and females. This finding is in contrast to the statement that the temporal trends were similar. However, due to the explorative nature of these post-hoc analyses as well as the problem of multiple testing, possible findings have to be interpreted with caution.
**Answer:** Thank you for your valuable opinions. We have provided it and marked it. (page 9, 10)
page 9: (Materials and Methods) The trend between males and females was compared by using a test of parallelism.
page 10: (Results) The tests of parallelism for the male and female trend lines in the Taiwanese population and US white population both were not significant (*p* = 0.456 and 0.133).

**Question** 2.
The SEER data show some differences across groups defined by age at diagnosis. For example, for all ethnicities, an APC of -1.9 was observed overall, while the estimates for the age groups were 8.6 for ages 0-19, -2.1 for ages 20-64 and -1.8 for ages ≥ 65. Estimates are also available for males and females. The variable age at diagnosis should also be available for the Taiwanese registry data. If so, the authors should add the estimates, as supplementary data. If data for groups by age at diagnosis are not available, please add a note on this issue.
**Answer:** The estimates had added in supplementary data, S3 Table. The APCs were 4.4 for ages 0-34, 2.6 for ages 35-64 and 4.0 for ages ≥ 65. For males, the APCs were 1.6, 2.7 and 2.9 for ages 0-34, 35-64, and ≥ 65, respectively. For females, the APCs were 8.2, 2.5 and 5.5 for ages 0-34, 35-64, and ≥ 65, respectively.
 Because the numbers of FL cases were almost zero for ages < 30 from 2008 to 2017, we stratified the cases into 3 age groups: 0-34, 35-64, and ≥ 65.

**Question** 3.
In table S2, results from joint analysis with data from Ko et al. are presented. How were the data for Table S2 merged in order to estimate the APC? Moreover, Ko et al. count lymphoma with ICD-O-3 codes 95973 and 96753 as follicular lymphoma (Ko et al., Table 1). Did the authors consider how this discrepancy between this and their own definition of follicular lymphoma could influence the results? The authors could, for example, present the number of patients with ICD-O-3 codes 95973 and 96753 in their own data set.
**Answer:** The age-standardized incidence rates (ASR) between 2002 and 2007 from Ko et al. (Table 2) and the ASR between 2008 and 2017 from our data were merged to estimate the APC by using the Jointpoint regression under the “constant variance assumption”.
 The ICD-O-3 code 9675/3 is not applicable since 2010. The cases with code 9597/3 were very rare in Taiwan, just one case was found in a medical center from January 1998 to December 2014 [1] and there were no case in other medical center between January 2001 and December 2010 in Taiwan [2], thus it would not affect the results in this study.
Reference:
1. Liu KL, Tsai WC, Lee CH. Non-mycosis fungoides cutaneous lymphomas in a referral center in Taiwan: A retrospective case series and literature review. PLoS One. 2020;15(1): e0228046. doi: 10.1371/journal.pone.0228046. PMID: 31978091.
2. Lee CN, Hsu CK, Chang KC, Wu CL, Chen TY, Lee JY. Cutaneous lymphomas in Taiwan: A review of 118 cases from a medical center in southern Taiwan. Dermatologica sinica. 2018; 36(1): 16-24. Doi: 10.1016/j.dsi.2017.08.004.

Minor Questions:
1. Please provide the formal definition of the annual percentage change.
**Answer:** We have provided it and marked it.
Page 8: APC is often used to characterize trends in disease rates, which assume the disease rates change at a constant percentage of the rate of the previous year by using this approach.
2. Please use the term “relative frequency of FL among the incident NHL cases” throughout the manuscript and in the tables and figures.
**Answer:** We have corrected it and marked it throughout the manuscript and in the tables and figures.
3. Please add the confidence intervals as well as the p-values to the last column of Table 1.
**Answer:** We have corrected it and marked it.
4. Caption to Table S2: reference number for Ko et al. should be 10 instead of 8.
**Answer:** We have corrected it and marked it for reference number in Supporting Information.
5. Introduction: add the unit (per year) to the statement “incidence rate of 3·5 per 100,000 persons in 2008–2017”
**Answer:** We have corrected it and marked i: incidence rate of 3·5 per 100,000 persons per year in 2008–2017
6. Materials and Methods, page 7: change “race” to “race/ethnicity” in the sentence “The SEER program records race as assigned by the North American Association of Central Cancer Registries.”
**Answer:** We have corrected it and marked it. (Page 3, 7, 9)
7. Results, page 10: the – declining – estimates for APC in whites, all as well as males and females, should be negative, i.e. -2.1, -1.8 and -2.4.
**Answer:** We have corrected it and marked it.
8. Caption for figure 1: add “incidence” to the word rate.
**Answer:** We have corrected it and marked it.

**Revierwer #2:**

**Reviewer #2:** (I) Summary

The authors of this manuscript explore recent trends in follicular lymphoma (FL) in Taiwan and United States (US) using cancer registry (Taiwan Cancer Registry and US SEER) data from 2008 to 2017 with the aim of elucidating incidence patterns in both populations. They provide recent data from both cancer registries on age-adjusted incidence rates, relative frequencies and annual percent changes (APC) of FL stratified by sex and race/ethnicity. Collected data show a steadily rising trend in FL incidence in Taiwan (+39% from 2008-17) as compared to declining incidences in US white population (-20% from 2008-17) and steady trend in Hispanic, black and Asian/Pacific Islander population. The authors discuss possible factors for changes in incidence (genetics, lifestyle, environment) and compared their newly gathered data on FL incidence to recent data from other Asian countries noting some similarities. They conclude that further etiological investigations in Asian countries are needed.
**Answer:** Thank you for your comment.

(II) Discussion of specific areas of improvement

1.) Abstract
- clear and concise, no comment

2.) Introduction
- no comment

3.) Materials and methods
- the US are ethnically quite diverse and how SEER handles ethnicity is explained in great detail, please add a short comment if ethnicity is an issue for the Taiwan Cancer Registry.
**Answer:** Individual data collected by Taiwan Cancer Registry doesn’t include patients’ ethnicity.

4.) Results
- 2nd paragraph, line 3: APCs and 95% CI are listed in Figures 1 and 2, while Table 1 provide only APCs, this should be corrected accordingly.
**Answer:** We have corrected it and marked it in Table 1.
-2nd paragraph, line 5: data of % change is provided for FL incidence in Taiwan (+39%), but no similar data provided for decrease in US white population, consider adding data.
**Answer:** We have provided it and marked it in 2nd paragraph, line 12: from 3·42 to 2·74 per 100,000 person-years between 2008 and 2017, representing a 20% decrease.
- S1 table: reference to study by Ko et al is 10 and not 8, please correct
**Answer:** We have corrected it and marked it for reference number in Supporting Information.
- S1 table: data for Korea12 and 15 shown with same color and symbol making it harder to read, consider correcting
**Answer:** We have corrected it and marked it: Korea_Lee, Korea_Kim.
- Table 1, whites, RF (%), APC: only variable with a joint point. Discuss with your statistician if an average APC could be calculated and added in the comment under the table or consider adding a comment why there are two number as opposed to all other APCs.
**Answer:** We have corrected it and marked it.
Page 17: In the whites, the relative frequency of FL among the incident NHL cases declined from 12.9% to 10.7%, the average APC was −1.8 (95% CI: −3.1% to −0.5%, p = 0.009).

5.) Discussion
- list possible limitations of this study and/or biases (for example: SEER data do not cover whole US population; incidence change in US white population cannot be generalized
**Answer:**
 We have corrected it and marked it.
 Page 21: The main limitation of this study is that SEER data does not cover whole US population, the incidence change in each racial population in US cannot be generalized. Another limitation of our study is that the FL data in Taiwan was retrieved from the TCR annual report, not Taiwan Cancer Registry Database. The number of FL cases by gender and age in the TCR annual report was defined by ICD-O-3 codes 9597/3, 9675/3, 9690/3, 9691/3, 9695/3, and 9698/3, that is different from the definition of FL in SEER. Although we could not know the number of cases in each ICD-O-3 codes, the ICD-O-3 code 9675/3 is not applicable since 2010. The cases with code 9597/3 were very rare in Taiwan, just one case was found in a medical center from January 1998 to December 2014 [41] and there were no case in other medical center between January 2001 and December 2010 in Taiwan [42], thus it would not affect the results in this study.

(III) Other comments
- very fluent reading, carefully edited text, no language issues.
**Answer:** Thanks for your comments.
