# Supplementary material for: Investigation of the incidence trend of follicular lymphoma from 2008 to 2017 in Taiwan and the United States using population-based data
Source: PLoS One. 2022 Mar 17;17(3):e0265543. doi: 10.1371/journal.pone.0265543 (PMC8929617; doi:10.1371/journal.pone.0265543)
Supplement: Attachment — Submitted filename: Response to Reviewers.docx [file pone.0265543.s004.docx]

To Editor and Reviewers:

Thank you very much for your detailed review of our manuscript **PONE-D-21-21835R1**, entitled “**Investigation of the incidence trend of follicular lymphoma from 2008 to 2017 in Taiwan and the United States using population-based data**”. We have provided a point-by-point revision or response to all your comments in balance. We provide the reasons and revisions in the following parts. For your convenience, all the changes are a highlight in red. During the revision, we also identify some mistakes in our article. We corrected them and marked in red.

We appreciate your valuable opinions. And thank you again for your kindly consideration for publication of our revision article.

Best regards.

Yu-Chieh Su MD, MSc.

Division of Hematology-oncology, E-Da hospital, Kaohsiung, Taiwan.

21, Jan, 2022

**Answer to Editor’s comments:**

- Please make sure that the limitation section of your paper indicates" that despite the SEER database, the Taiwan Cancer registry doesn’t include patients’ ethnicity".
- Please explain how the study handled missing data.
- Please answer comments made by reviewer#3

**Answer:** Thank you for your reminder.
1. The TCR annual report does not include patients’ ethnicity have been confirmed.
2. The follicular lymphoma data in Taiwan and US available from the Taiwan Cancer Registry annual report of the Health Promotion Administration, Ministry of Health and Welfare, Taiwan and the US National Cancer Institute’s SEER 18 program, respectively. The cancer registry annual report based on the databased established by the Taiwan Cancer Registry which case completeness rates greater than 97%. The Taiwan Cancer Registry implemented rigorous quality control protocols to validate and verify cancer diagnoses, including submission of tissue pathology reports and random sampling of claims and diagnosis reports. It is considered a complete and accurate registry. The SEER program records race as assigned by the North American Association of Central Cancer Registries, which is only comprehensive source of population-based in the US and have a case completeness rate greater than 98%.

**Answer to Reviewers’ comments:**

**Revierwer #2:** The authors have adequately addressed all questions from the previous round of review posted by both reviewers. The manuscript has been improved in the Materials, Results and Discussion sections, tables and charts have been revised according to recommendations. At this point I have no further comments.

**Answer:** Thank you for your comments.

**Revierwer #3:**

1- Why the authors compared the incidence of FL in Taiwan and US? What is the rationale behind it?
**Answer:** We want to compare the trends for incidence rates of follicular lymphoma in Taiwan and other Asian ethnic groups in recent years, but we cannot obtain these data from Japan, South Korea and other Asian countries. Therefore, we compared the data for Taiwanese and Asian ethnic groups in US from SEER database, and also observed the trends for incidence rates of FL for other ethnic groups in US from SEER database.

2- It is highly recommended to provide the report based on the guidelines such as STROBE or the following: https://www.ncbi.nlm.nih.gov/books/NBK208602/.
The authors should provide some subheadings in the methods to cover the main following questions regarding registry data:
• Study purpose: Were the objectives/hypotheses predefined or post hoc?
• Patient population: Who was studied?
• Data quality: How were the data collected, reviewed, and verified?
• Data completeness: How were missing data handled?
• Data analysis: How were the analyses chosen and performed?
**Answer:** Thank you for your comments. We have provided it and marked it in red (Page 6-9).

3- In the abstract the authors stated that “Our findings suggest that modifiable risk factors may be important determinants of the diverse trend of FL, especially in Taiwan.” This conclusion does not seem logical since the authors did not examine the role of modifiable risk factors in this study. Conclusions should be consistent with the findings of the study.
**Answer:** Thank you for your comments. We have deleted this conclusion in the abstract.

4- It is recommended to quantify the association of age, period, and cohort using age–period–cohort models.
5- Reporting incidence rate ratios (IRRs) for assess the cohort effect is recommended.
**Answer:** Thank you for your comments. Since the number of FL cases in Taiwan were showed by age in 5-year age groups from the TCR annual report and the study period was from 2008 to 2017, the number of consecutive 5-year periods was just two, therefore, the age-period-cohort model was not applicable. We will use the age-period-cohort models in the future studies.
